# Curcumin Delivery Mediated by Bio-Based Nanoparticles: A Review

**DOI:** 10.3390/molecules25030689

**Published:** 2020-02-06

**Authors:** Mahshid Moballegh Nasery, Banafshe Abadi, Delaram Poormoghadam, Ali Zarrabi, Peyman Keyhanvar, Hashem Khanbabaei, Milad Ashrafizadeh, Reza Mohammadinejad, Shima Tavakol, Gautam Sethi

**Affiliations:** 1 Student Research Committee, Kerman University of Medical Sciences, Kerman 7619813159, Iran; nasery278@gmail.com (M.M.N.); banafshe.abadi@yahoo.com (B.A.); 2 Department of Toxicology & Pharmacology, School of Pharmacy, Kerman University of Medical Sciences, Kerman 7616911319, Iran; 3 Nanomedicine Research Association (NRA), Universal Scientific Education and Research Network (USERN), Tehran 7616911319, Iran; 4 Department of Medical Nanotechnology, Faculty of Advanced Sciences & Technology, Pharmaceutical Sciences Branch, Islamic Azad University, (IAUPS), Tehran 1916893813, Iran; parand.pdrm@yahoo.com; 5 Sabanci University Nanotechnology Research and Application Center (SUNUM), Tuzla, Istanbul 34956, Turkey; alizarrabi@sabanciuniv.edu; 6 Research Center for Pharmaceutical Nanotechnology, Faculty of Pharmacy, Tabriz University of Medical Sciences, Tabriz 5165665811, Iran; regenerative.md@gmail.com; 7 Department of Medical Nanotechnology, School of Advanced Medical Sciences, Tabriz University of Medical Sciences, Tabriz 5165665811, Iran; 8 Medical Physics Department, School of Medicine, Ahvaz Jundishapur University of Medical Sciences, Ahvaz 6135715794, Iran; khanbabaie.mph@gmail.com; 9 Department of Basic Science, Faculty of Veterinary Medicine, University of Tabriz, Tabriz 5166616471, Iran; dvm.milad73@yahoo.com; 10 Neuroscience Research Center, Institute of Neuropharmacology, Kerman University of Medical Sciences, Kerman 7616911319, Iran; 11 Cellular and Molecular Research Center, Iran University of Medical Sciences, Tehran 1449614525, Iran; 12 Department of Pharmacology, Yong Loo Lin School of Medicine, National University of Singapore, Singapore 117600, Singapore

**Keywords:** curcumin, cancer, nanocarriers, biopolymer, exosomes

## Abstract

Todays, nano-pharmaceutics is emerging as an important field of science to develop and improve efficacy of different drugs. Although nutraceuticals are currently being utilized in the prevention and treatment of various chronic diseases such as cancers, a number of them have displayed issues associated with their solubility, bioavailability, and bio-degradability. In the present review, we focus on curcumin, an important and widely used polyphenol, with diverse pharmacological activities such as anti-inflammatory, anti-carcinogenic, anti-viral, etc. Notwithstanding, it also exhibits poor solubility and bioavailability that may compromise its clinical application to a great extent. Therefore, the manipulation and encapsulation of curcumin into a nanocarrier formulation can overcome these major drawbacks and potentially may lead to a far superior therapeutic efficacy. Among different types of nanocarriers, biological and biopolymer carriers have attracted a significant attention due to their pleiotropic features. Thus, in the present review, the potential protective and therapeutic applications of curcumin, as well as different types of bio-nanocarriers, which can be used to deliver curcumin effectively to the different target sites will be discussed.

## 1. Introduction

In ancient cultures, the plants played a crucial role in providing food, spices, and medication to the human population [1,2,3,4]. A number of nutraceuticals have displayed significant health benefits, in modern era among which curcumin has been extensively studied for its pleiotropic therapeutic actions [5]. The rhizome turmeric derived from *Curcuma longa Linn* consists of various curcuminoids, including curcumin, demethoxycurcumin and bisdemethoxycurcumin [6,7]. Among these curcuminoids, curcumin is the most abundant polyphenolic compound in turmeric, which is widely used as a spice and flavoring agent in the food [8]. It was discovered about two centuries ago and has a slightly bitter taste, peppery flavor, and smell like mustard with yellow color [8]. Pharmacologically, curcumin is safe and can mitigate tumor initiation as well as metastasis in breast, colon, pancreatic, oral and several other cancers [9,10,11,12]. As mentioned earlier, curcumin has shown remarkable anticancer activities by affecting diverse molecular targets. It can lead to an increased expression of Bax and p53 (pro-apoptotic proteins), suppression of vascular endothelial growth factor (VEGF) and hypoxia-inducible factor 1-alpha [HIF-1α] (angiogenesis factors), reduction of the pro-inflammatory responses, induction of autophagy and improvement of drug efflux in drug resistance cancer cells [13,14,15,16,17]. It also appears to be a promising agent for the treatment of brain disorders, cholesterol, and endothelial dysfunctions and can serve as a potent anti-inflammatory and anti-viral agent as well [18]. Furthermore, there is a report on the reduction of opioid tolerance by curcumin through the inhibition of the activity of Ca2+/calmodulin-dependent protein kinase II α. This kinase has been found to be critical for the opioid tolerance [19]. Notably, curcumin at an optimized dose has low toxicity and is inexpensive, which makes it an ideal herbal for clinical applications [20]. Notwithstanding, the poor bioavailability of curcumin may limit its application in clinical administrations [21]. However, the low concentrations of curcumin are sufficient for its biological activity and they may apply as a supplement in multiple targets therapy in combination with other medications to improve their therapy efficacy [22]. To date, several curcumin carriers have been synthesized as a drug delivery system using viruses, liposomes, magnetic nanoparticles (NPs), ultrasound microbubbles, etc. [23,24]. It is important to mention that the size of nanocarriers may also affect the therapeutics effect of their cargo [25]; for example, Tavakol et al. showed that the size of a carrier changes the therapeutic effect and biocompatibility of curcumin [26]. Moreover, the chemical and physical nature of carriers may induce side effects, including organ toxicity and/or immune responses [27,28]. Moreover, carriers may exhibit non-uniform particle size distribution, particle agglomeration, non-specific uptake, and rapid clearance from the blood [28,29,30]. Liposomes are one of the most popular carriers used in drug delivery [31,32,33]; however, in some cases, they suffer from fast elimination from the blood circulation, physical and chemical instability, aggregation, fusion, degradation, hydrolysis and phospholipid oxidation [29,34].

Recently, different types of biopolymers have been introduced with the capability of being used as carriers for delivering curcumin among them are chitosan, starch, zein, alginate, silk, etc. The distinguishing features of these nano-systems like biodegradability, biocompatibility, eco-friendliness, and a wide range of commercial applications have made them as an ideal candidates for the drug delivery applications [35]. These types of polymers can incorporate drugs through two main methods; desolvation process (for proteins) and nanoprecipitation (for polysaccharides) in different forms of the hydrogels, single biopolymers, and complex biopolymers [36]. 

The other types of nanocarriers are biological carriers like exosomes that are secreted by most cells of the body and provide a favorable drug delivery efficacy [30]. Exosome diameter is in the range of 30–120 nm, and they can be derived from the extracellular fluids such as blood, urine, amniotic fluid, saliva, and cerebrospinal fluid. Exosomes can carry many molecules like RNA, proteins, and lipids [37]. Interestingly, encapsulation of curcumin into the exosome can improve curcumin solubility, stability, and it’s in vitro and in vivo bioavailability [38]. As mentioned earlier, the therapeutic efficacy of curcumin generally requires low to moderate concentrations, which are attainable by encapsulation of curcumin into exosomes. Curcumin-encapsulated exosomes provide high plasma concentration along with low toxicity and immune response induction [18]. In addition to their anti-aging and antioxidant properties, they can be effective against venom activities, protozoal and microbial contamination, inflammatory responses, angiogenesis procedure, and tumor suppression through the presence of exosomes [39]. The present review is a short description of curcumin, a widely used polyphenol exhibiting anti-cancer and anti-inflammatory activities and different biopolymeric as well as biological nanocarriers that can be used to facilitate substantial improvement in its bioavailability for therapeutic applications.

## 2. Application of Curcumin for Cancer Therapy

Cancer is regarded as a major global public health problem [40,41,42]. The rate of cancer mortality is increasing worldwide from 2012 to 2015. In 2012, cancer caused 8.2 million deaths that rose to 8.8 million deaths in 2015. Notably, it is predicted that global cancer causes will be over 20 million new cases by 2025 [43]. There are different types of therapeutic options for cancer therapy including radiotherapy, immunotherapy, surgery, chemotherapy, etc., among them, chemotherapy is known as the most abundant method used for global cancer treatment [44,45]. Interestingly, SarinaMedTrip a famous international health tourism company utilizes the latest technologies in the world to treat cancer patients [46]. However, the chemical drugs used for chemotherapy exhibit several side effects such as nausea, vomiting, hair loss, leukopenia, diarrhea, hepatotoxicity, nephrotoxicity, etc. 

Recently, natural agents have received lots of attention due to their diverse pharmacological activities [47,48,49,50,51,52]. They are derived from plants with the ability of cancer prevention or treatment possibly with reduced side effects [53,54,55,56,57,58,59,60,61]. Curcumin can exhibit a wide range of pharmacological potential [62,63,64] including anti-inflammatory, anti-oxidant, anti-proliferative, chemo-sensitizing, cell cycle arrest, and can display apoptotic potential against various cancer cells, such as colorectal, breast, pancreatic, and colon cancers [65] that makes it of great interest in cancer prevention and therapy [66,67]. Notably, IC50 of curcumin in healthy cells is significantly higher than cancer cells [68]. In other words, it is well tolerated and used as a spice, coloring agent, and supplement up to a dose of 12 gram/day [69].

Notwithstanding, it is not entirely understood how curcumin is responsible for cell protection [68]. For example, the anti-inflammatory potential of curcumin plays a critical role in cancer therapy [70]. Moreover, curcumin inhibits the activity of transcription factor NF-κB and prevents its transmission to the nucleus. Therefore, it can lead to the down-regulation of various inflammatory and oncogenic genes [6]. Besides its anti-inflammatory potential, there are some reports on the dual role of curcumin as a reactive oxygen radical scavenger and producer; however, it appears that curcumin through the reactive oxygen radical production can also induce apoptosis in cancer cells [68]. It is noteworthy, that curcumin can also affect multiple cell signaling pathways to negatively affect cancer cells, for example, it inhibits VEGF and suppresses VEGF receptor-2, fibroblast growth factor 2, matrix metalloproteinases, 2 and 9 etc [66].

Curcumin has shown its positive effects both in vivo and in vitro models [71,72]. It has anti-proliferative potential in a concentration-dependent manner [70] and is useful in combination with anticancer agents; for example, its cytotoxicity in prostate cancer cells reduces the survival of these cells [69]. It also affects cancer stem cells and shows anti-metastatic activity [43] and can be well tolerated at doses near 8000 mg/day [73]. In one report, the effect of orally consumed curcumin on the apoptosis of colon tumors was investigated. Their results indicated that curcumin, as a nutritional supplement can enhance apoptosis and inhibit tumor progression [74]. Based on another study, it was concluded that curcumin can also mitigate the progression of hepatoma cells and metastases, which are driven by EMT-induced through TGF-β1. The authors suggested that curcumin suppresses the phosphorylation of Smad2 through the TGF-β1 over-expression that resulted in Snail down-regulation [70].

## 3. Challenges Associated with Curcumin Delivery

Although a large number of nutraceuticals have shown substantial anti-cancer potential by inhibiting various oncogenic molecular pathways in cancer cells and preclinical studies, they have failed in clinical trials owning to their low bioavailability [32,33,75,76]. However, curcumin has shown significant benefits in clinical settings and it has been found to be non-toxic and fairly well-tolerated, however its solubility, and bioavailability may limit its usage in patients [7]. In other words, clinical use of curcumin in the patients may be limited due to its low bioavailability, short half-life in plasma, low solubility in water, and low stability [8,41,75,77] and it may necessitate high dose intake to achieve optimal therapeutic effects. Interestingly, several of the important anti-carcinogenesis effects of curcumin may be related to its significant ability to modulate transcription factor NF-κB activation, however, it suffers from poor bioavailability and solubility. To overcome this obstacle, Elias et al. prepared curcumin in guar gum tablet and enhanced curcumin solubility and bioavailability effectively [78].

The second problem associated with its application is its rapid metabolism and enterohepatic circulation that necessitates the higher doses of curcumin to induce pronounced efficacy [41,69]. Moreover, fast clearance from the bloodstream through the phagocytosis and reticuloendothelial system is another major issue [75]. There are many investigations related to the enhancement of solubility and stability of curcumin, for example, encapsulation of curcumin into lipid based carriers, conjugation with nanoparticles, etc [26,41]. In the rest of the review, we have discussed these diverse methods and highlighted the various procedures to overcome these drawbacks and increase curcumin efficacy in the patients.

## 4. Biopolymer Nanoparticles (NPs)

Biopolymeric particles are colloidal structures that are assembled from one and multiple types of biopolymer molecules and can be utilized as carriers for bioactive compounds [79,80,81,82]. Biopolymeric particles can be used to improve the stability of bioactive molecules from the bio-degradation, enhance their absorption, and deliver them to the target sites [83]. Notwithstanding, they can act as a controlled release system for the sustained and controlled release of their ingredients [84]. Biopolymeric particles are classified based on the method of preparation (inclusion complex), types of nano dimensions (nanofiber, nanosphere, etc.) [85,86,87] and chemical components (chitosan, gelatin, etc.) [88,89,90]. As shown in Table 1, there are several investigations address the methods of nanocurcumin preparation and its biological efficacy (in vitro and in vivo). 

### 4.1. Protein-Based Biopolymers

#### 4.1.1. Albumin

Albumin is one of the main proteins of plasma with high stability, biodegradability, non-immunogenicity, and biocompatibility. Moreover, it has lots of binding sites in its matrix that make it an ideal platform for drug loading [77]. Human serum albumin (HSA) is one of the smallest proteins in blood plasma, which can transport therapeutics in the bloodstream. Furthermore, albumin-binding proteins are over-expressed on the surface of endothelial cells in tumor vessels, and therefore, HSA can be accumulated in the tumor sites through transcytosis across continuous endothelium [117,118]. Since the low water solubility of curcumin and its poor bioavailability may result in the decrease of therapeutic efficacy, encapsulation of curcumin into a water-soluble albumin carrier can be useful to overcome these obstacles [119]. For instance, in a recent study, curcumin was encapsulated into the HSA particles to achieve a redox-responsive release of curcumin. According to the results, the release of curcumin was considerably increased in the presence of glutathione in the physiological pH (7.4) of the body or acidic pH (5.5) of the tumor environment during the 48 h (57% and 70% of the loaded drugs, respectively). Moreover, HSA particles improve cellular uptake of curcumin compared to free curcumin that leads to increase anti-cancer efficacy [77]. In another study, curcumin was encapsulated into the bovine serum albumin (BSA)-conjugated dextran NP resulting in increasing the size from <200 nm to 512 nm. Notably, NPs showed high stability against pH and temperature changes with high cellular antioxidant activity [91]. Moreover, Saleh et al. deciphered the potential of human epidermal growth factor receptor 2 (HER2) aptamer-decorated human serum albumin NPs loaded with curcumin (281 nm) on HER2 overexpressing breast cancer cells. They showed that curcumin conjugated Apt-HSA/CCM NPs significantly augmented aqueous solubility and cellular uptake. Cytotoxicity was also elevated crucially on the SK-BR3 cell line compared with unconjugated counterparts [120]. The optimal size of NPs is fundamental to achieving the maximum cellular uptake. A more recent examination with curcumin cross-linked HSA NPs in a size range of 25–250 nm conducted by Das et al. showed that 125 nm particles found to have noticeable cellular uptake and cytotoxicity on A549 cell line compared to the free drug [92].

#### 4.1.2. Zein-Based NP

Zein is an alcohol-soluble protein that is the major protein in the corn kernels and is made up of a high level of non-polar amino acid. This feature along with other properties like high biocompatibility and stability makes it an ideal polymeric carrier for encapsulation of hydrophobic molecules like curcumin [121,122]. The zein-hyaluronan NPs loaded with curcumin were prepared by the anti-solvent co-precipitation method. Results indicated in curcumin loaded into these carriers showed high stability against light and resulting in a controlled release system in simulated gastrointestinal digestion [100]. Therefore, zein NPs can be used to encapsulate curcumin and enhance curcumin efficiency [123,124]. 

Zein can also be used in combination with other types of reagents, for example, electrostatic complexes consisting of a protein (gelatin) and a polysaccharide (alginate) were prepared to coat and stabilize zein NPs loaded with curcumin [93]. Moreover, hyaluronan was also used as a coating agent to achieve high encapsulation efficiency, loading capacity, greater stability, and controlled release system during gastrointestinal digestion [100]. Moreover, the potential of a zein-caseinate composite as a carrier has also been investigated. The results showed high re-dispersibility in water, high encapsulation efficiency, bioavailability and anti-oxidant property of curcumin [94]. Furthermore, to improve the stability of these NPs, pectin was used as a carbohydrate-based coating. Pectin coating not only increased the loading capacity of NPs to encapsulate curcumin but also promoted a sustained release of curcumin under gastrointestinal conditions [95].

Chen et al. synthesized a combination therapy system of a layer-by-layer NPs consisting of curcumin entrapped into Zein NPs. The NPs were covered by a quercetagetin shed and hyaluronic acid (HA). The size of particles was 231 nm and showed convincing physical stability and slow rate release as well as a decreased light and thermal degradation [122]. In another study, curcumin was loaded into Zein NPs and then they were double coated with sodium caseinate and sodium alginate to stabilize the NPs structure (~70 nm). Aqueous solubility, drug-controlled release, photochemical stability, and antioxidant scavenging activities were promoted significantly as compared to the unbound curcumin. Much of recent literature on loading curcumin into nanoscale carriers are perpetrating intended for the goal of improved oral drug delivery to the gastrointestinal tract [96]. Zein also was used in the form of nanogel which was cross-linked with HA, as targeting agent, for curcumin delivery, that showed improvement in the in vitro and in vivo pharmaceutical activity of the drug [125]. In another study, zein and ι-carrageenan were used as core-shell NPs to prepare a photo and thermal stable carrier for co-delivery of curcumin and piperine [126]. 

#### 4.1.3. Silk-Based NPs

Silk fibroin is a type of biopolymers with different secondary structures (α-helix, β-sheets, coil, etc), which are used in different constructions like film, hydrogel, matrix, fiber, NPs, etc. [127]. It seems that due to remarkable properties such as high stability, negatively charged, and low toxicity, silk NPs attract much attention to be used as a carrier for curcumin. Notably, the curcumin-loaded silk fibroin NPs showed considerable cytotoxicity against neuroblastoma cells [7]. In another study, curcumin was loaded into magnetic silk fibroin core-shell NPs as an external magnet for cancer targeting. The composite showed high cytotoxicity and cellular uptake against triple negative breast cancer MDA-MB-231 cells [128]. Crivelli et al. revealed that curcumin encapsulated into silk fibroin NPs (71 ± 10 nm) enhances in vitro antioxidant, anti-inflammatory activities, and cell viability. The remarkable anti-inflammatory properties of curcumin have been established as a promising strategy for osteoarthritis treatment [97].

In a recent experiment, oral administration of curcumin- loaded silk particles with a wide size range of 229–2286 nm carried out for evaluating the correlation between particle size and bioavailability in rat models. Larger silk particles (with a size of about 800 nm) exhibited longer plasma half-life and slower release rate, while smaller silk particles (with a size of about 200 nm) indicated higher bioavailability and Cmax. Bioavailability was 5-fold and 17-fold higher than free drug in 800 and 200 nm particles, respectively [98]. In another study, a silk nanofiber membrane is produced to co-load curcumin and 5-fluorouracil. The prepared membrane had about 100–200 nm size that released its cargo during a steady and consisted method and thus, it can use as an ideal anti-cancer delivery system [129].

#### 4.1.4. Other Protein-Based NPs

Different proteins with various sources have been used as a carrier for curcumin [130,131,132]. For instance, in 2019, Radix Pseudostellariae protein (RPP)-based NPs were fabricated to loaded with curcumin. The curcumin-loaded nanocomplexes with a size of 100 nm showed considerable thermal stability and high light stability [133]. In another study, proso millet protein was utilized as a carrier to increase curcumin therapeutic efficacy. The millet-curcumin has a spherical shape with 300 ± 50 nm size and an extensive range of drug loading, which was attributed to the millet extraction method [134]. 

Rice bran waste is rich in proteins and other essential compounds such as lipids, vitamins, and trace minerals. In a recent study, the rice bran albumin (RBA) was derived from the rice bran waste to be used as a carrier for curcumin. RBA was blended with chitosan and formed NPs through the self-assembly method. The results showed RBA–chitosan NPs improves the solubility and high entrapment efficiency of curcumin (about 93.56%). The RBA–chitosan NPs showed low bio-degradability in gastric conditions along with satisfied biodegradability and high cytotoxicity against cancerous cells compared to free curcumin that confirmed its usefulness as a drug delivery carrier [99]. Recently, a dual triggerable release nanosystem was prepared to encapsulate curcumin. In this study, novel hydrogel NPs based on fibrous structural proteins (keratin) and thermo-responsive copolymers (Pluronic) have been fabricated to achieve redox and temperature-responsive release of curcumin, and the results were promising. In fact, by changing the temperature from 25 °C to 37 °C, the size of the drug-loaded nanocarrier was also reduced from 165 nm to 66 nm that revealed the drug release property of the nano-system due to its thermo-responsive property [135]. Moreover, Li et al. loaded curcumin into solid and hollow Kafirin (a protein found in sorghum grain) and then coated with layer by layer deposition of Dextran sulfate/Chitosan. Particle sizes were around ~60 and ~125 nm for hollow and solid NPs, respectively. Despite their smaller size, hollow NPs presented greater encapsulation efficiency. The release rate in both hollow and solid structures was higher than free curcumin, while hollow NPs manifested lower release compared to solid ones [136].

### 4.2. Polysaccharide NPs

Polysaccharides are composed of repeated monosaccharide units joined bound by glycosidic bonds. Due to their advantages, such as high stability, biocompatibility, and biodegradability, they can be used for different applications in biomedical field [95].

#### 4.2.1. Chitosan

One of the most commonly used polysaccharides for the preparation of nanocarriers is chitosan [137]. Chitosan is a linear cationic heteropolymer derived by the partial deacetylation of natural chitin. It was introduced by Charles Rouget in 1859 for the first time and can promote cell membrane permeability, and thus, enhance absorption across intestinal epithelia [138,139]. It is a positively charged polymer with d-glucosamine and *N*-acetyl-d-glucosamine units and excellent properties like biocompatibility, biodegradability, low immunogenicity, and antibacterial activity that make it an ideal carrier for drug delivery purposes [140,141]. 

It is widely used as a carrier for delivering curcumin, alone or in combination with different types of components, for example: the polyelectrolyte complexation of positively charged chitosan and negatively charged acylated cruciferin, was used for the curcumin entrapment. In vitro control-release studies showed the controlled release of curcumin using simulated gastro-intestinal fluids; however, the curcumin NPs showed non-toxicity against Caco-2 cells [102]. Curcumin- loaded chitosan NPs (167–251 nm) exhibited enhanced entrapment efficiency, enhanced transdermal permeation, improved drug release, and high cell viability in transdermal drug delivery [103].

In a recent study, curcumin loaded chitosan NPs with a size of about 200 nm were prepared to be used as a chemotherapeutic agent for lung cancer. The results of this research confirmed a significant improvement in the cytotoxicity of the drug-loaded nanocarrier and also introduced this type of nanosystem as an oral supplement against environmental carcinogenesis [142]. In another study, curcumin was loaded into a pH-responsive nanocapsule that is composed of mesoporous silica and chitosan for using against the U87MG glioblastoma cancer cell line. The nanocarriers had about 88.1 ± 4.76% encapsulation efficiency and curcumin release was sustained-in acidic pH (~42.72% during 96 h). Moreover, this form of curcumin encapsulation could reduce the IC50 of it from 15.2 μg/mL for the free drug to 5.21 μg/mL for the loaded one [143]. Razi et al. prepared a formulation of genipin cross-linked caseinate chitosan NPs for curcumin delivery, that had about 250 nm size and could increase the stability and anti-cancer property of the curcumin [144]. 

#### 4.2.2. Alginate

Alginate is a negatively charged biodegradable polysaccharide, which is composed of 1–4 linked α-l-guluronic (G) and β-d-mannuronic (M) acid residues and can exclude from gulfweed, bacteria or seaweed of brown algae [145]. It is a linear polysaccharide that has significant advantages like high mucoadhesiveness, aqueous solubility, biodegradability, pH sensitivity, and biocompatibility [146]. Sorasitthiyanukarn et al. recently reported that chitosan/alginate NPs loaded with curcumin diethyl diglutarate (215 nm) promote sustain release, digestibility, bioaccessibility, physicochemical stability, and cellular uptake of curcumin [104]. In a recent study, polyethylene glycol (PEG) was grafted to polyethyleneimine (PEI) to form a PEG-b-PEI (mPPS). Then, it was coupled with folic acid as a ligand to target tumor cells that display an increased expression of folate receptors. Furthermore, the FA-PEG-b-PEI carrier was assembled with curcumin loaded alginate NPs. Gomez et al. developed a novel and efficient photodynamic therapy system for psoriasis therapy. In this study, curcumin-loaded chitosan/alginate NPs were found to repress the hyperproliferation of TNF-α induced HaCaT cells by using blue LED light [106]. Xu et al. prepared a nano-emulsion based alginate hydrogel beads for curcumin encapsulation. The nanocarrier had 24 nm size with 99% drug entrapment efficiency and pH-responsive drug released pattern [147]. 

#### 4.2.3. Starch

One other type of biopolymers is starch that is found abundantly in different parts of plants and is structured by interactions between glucose monomers in two forms, branched amylopectin (70%–80%) and linear amylose (20%–30%). This is a reactive biopolymer that can be modified with various methods and used for different applications, especially it can be applied as a drug delivery nanosystem for curcumin [148]. In this regard, there is a study conducted by Acevedo-Guevara et al., in which the acetylated starch extracted from green bananas used as a carrier for curcumin with an average size of 250 nm. Results showed that acetylated nanocarriers had higher encapsulation efficacy and controlled release potential than natural forms [107]. Furthermore, octenyl succinylated cassava starch NPs loaded curcumin (10–50 nm) showed exclusive water solubility, bioavailability, control release, cellular uptake, and anti-cancer potential [108].

The micellar structure of curcumin starch was prepared through the self-assembly of curcumin conjugated with hydrophilic hydroxyethyl starch by using an acid-labile ester linker. This new type of nanocarrier had a uniform size less than 100 nm with a dramatic enhancement in the solubility of curcumin, along with increasing its storage stability, and also pH-responsive release profile [149]. Starch was also used in the form of composite with other materials for cancer prevention and treatment with curcumin [150,151]. For example, Athira et al. prepared a curcumin loaded starch-poly(vinyl alcohol) nanocomposite with the size of about 50–200 nm that improved its anti-cancer activities [152]. In a new study, a smart nanogel based on the covalent interactions between carboxymethyl starch and chitosan hydrochloride was prepared, and curcumin was loaded in it with high entrapment efficiency (89%–95%). Additionally, it showed a sustained released profile that was responsive to pH changes [153].

#### 4.2.4. Cellulose

Cellulose is a type of β-glucan polysaccharide consist of glucose monomers that are attached through (1,4)-β- linkage to each other. This is a type of biopolymer that is widely found in the cell wall structure of plants, bacteria, algae, and fungi [154]. This is a natural polymer with characteristics like low-cost, biocompatibility, low density, hydrophilicity, and suitable modification that make it an ideal candidate for drug delivery application [155]. 

The application of this biopolymer for delivery of curcumin as an anti-cancer drug is discussed in different studies; for example, cellulose nanocrystal film loaded with curcumin was synthesized as an antimicrobial nanocarrier in the diabetic rat model. In vitro experiments showed a prolonged release of drugs for 36 h accompanied by the lack of burst effects. However, in vivo investigations demonstrated a significant decline in wound size and inhibition of bacterial growth. Sebaceous glands and hair follicles were also repaired. This new wound dressing could be an alternative for heavy metal substitutes such as silver ions [109]. In an experiment by Kanagarajan et al., pH-sensitive NPs were fabricated, in which MnFe_2_O_4_ curcumin loaded NPs coated with carboxymethyl cellulose by glutaraldehyde crosslinking (27 nm). These superparamagnetic biocompatible NPs can facilitate the release of cargo at endosomal acidic conditions (pH 5.5) [156]. Kang et al. hybridized curcumin loaded nanostructured lipid carriers (~500 nm) with cellulose nanofiber films for the treatment of imiquimod-induced psoriatic in mice model. Significant deposition of curcumin to the epidermis in addition to skin hydrating booster effect of cellulose nanofiber film resulted in relief of psoriatic symptoms and reduction of pro-inflammatory cytokine levels [110]. Moreover, Ngwabebhoh et al. synthesized curcumin encapsulated pickering NPs with an average size of 150 nm to improve curcumin bioavailability. The results suggested a high encapsulation efficiency and sustained release of curcumin, thereby indicating an enhanced antimicrobial and anticancer potential of drug [111]. 

## 5. Exosomes

Exosomes are bilayer membrane nano-sized vesicles derived from endosomal compartments with an average diameter of 30 to 100 nm [157]. Its biogenesis is a tightly controlled method of inward budding from the limiting membrane of multivesicular bodies (MVBs) [158]. The internal contents are released into the extracellular space in the form of “exosomes” when MVBs are fused to the plasma membrane. Exosomes can be found in body fluids such as blood, plasma, urine, saliva, amniotic fluid, synovial fluid, malignant ascites, and pleural effusions [159]. However, they can be produced by most cells, including B cells, T cells, dendritic cells, macrophages, neurons, glial cells, the most tumor cells, and stem cells. It seems that most cell types from normal cells to unhealthy cells release exosomes. Notably, exosomes based on their phenotypes and body fluids secrete diverse bioactive molecules such as proteins, lipids, and nucleic acid. In other words, exosomes participate in the transferring of signal transduction and intercellular communication from the main cell to the receptor cell in the form of proteins, mRNAs, ncRNAs, and miRNAs. Moreover, exosome contents are protected from the destruction of extracellular factors that it guarantees their half-life and biological activity enhancement [160]. Exosomes are made up of different types of lipids, such as cholesterol, sphingolipids, phosphoglycerides, ceramides, and saturated fatty acid chains. However, they have proteins such as transport proteins, heat shock proteins, proteins associated with multi-vesicular body biogenesis, and tetraspanin. They also have nucleic acids in the form of miRNA, mRNA, and other non-coding RNAs [124]. 

Thus, exosomes play an essential role in intercellular communication without direct cell-to-cell contact [161]. Although exosomes are not NPs derived from the nanotechnology due to its non-mankind nature, they may act as a nanocarrier owing to their particle diameter. Therefore, exosome’s particle size resulting in deep penetration into the tissues [162] and overcoming barriers such as the blood-brain barrier and the deformable cytoskeleton. Notably, they have slightly negative zeta potential that guarantees their long circulation [163]. In addition, some exosomes are capable of escaping from the immune system and have shown low immunogenicity and high stability in the blood, which prolongs the circulation of the drug within the body [164]. Furthermore, exosomes can be employed to load a variety of small bioactive molecules as a nanocarrier such as paclitaxel, doxorubicin, and curcumin, as well as peptide- or protein-based therapeutics. In addition, the loading of exosomes with a genetic material such as siRNA has also been reported [165]. To sum things up, due to their naturally biocompatible characteristics, exosomes are a promising candidate for clinical applications.

### 5.1. Advantage and Disadvantage of Exosomes

NPs seems to be promising for drug delivery of biomedical and pharmaceutical agents [77]. For example, liposomes control the release of drugs with an optimal synergistic molecular ratio and increase the concentration of the drug at the tumor site through the enhanced permeability and retention (EPR) effect. Although. liposomes reduce toxicity and side effects in the normal cells [41], the use of liposomes is limited due to some disadvantages such as poor stability, short half-life, rapid removal by the reticuloendothelial system, intracellular interactions or absorption, lipid particle growth, tendency to gelation and their intrinsic low incorporation rate due to the structure of the solid lipid [29,166]. Unlike conventional NPs such as liposomes and polymeric NPs, exosomes are naturally stable [124,167,168] and potentially diminish endosomal pathways and lysosomal damage while carrying their cargo directly into the cytoplasm. To have a comparison to cell therapy, exosomes are easier stored and reduces risks [30]. One of the remarkable benefits related to exosome is their permeability to damaged tissues and tumor sites and the ability to cross the blood-brain barrier (BBB) [124,169]. Therefore, exosome is an effective carrier to overcome the problems associated with drug delivery to the brain in clinical trials [170]. Besides, they are stable in the blood that allows them to remain in the body for a long time under various conditions. It is worth mentioning that exosomes have a by-layer shape with the hydrophilic feature in both surface and hydrophobic features in interlayer space that makes them suitable for both lipophilic and hydrophilic drug delivery [30]. 

There have been investigation related to natural cells such as bacteria, viruses, and eukaryotic cells [171]. Compared to liposomes and virus-based delivery systems, the immunogenicity of exosomes is very low [30], which makes them a suitable system for in vivo applications due to their more biocompatibility. Another benefit of exosomes is that therapeutic exosomes can be isolated from the patients and used for delivery and personal injections of the drugs. Exosomes that are provided from a suitable source are essential for conducting load loads to targeted tissues because the fat and compounds present at the cell surface are exclusive to these exosomes, and it is important to maintain this feature [169]. A critical problem in clinical studies is the lack of an optimal method for obtaining pure exosomes. This is primarily due to the relatively low amounts of released exosomes by mammalian cells. Therefore, a long-term source of exosomes with excellent characteristics and effective separation method is needed to obtain a large number of pure exosomes [30]. 

Another challenge with exosomes as a nanocarrier is their drug loading capacity [172]. Therefore, increasing the loading capacity of cargo and non-destructive targeted capabilities is crucial for favorable drug delivery. Finding more optimized approaches to manipulate the structure and function of exosomes that may promote their clinical applications is therefore vital [30].

### 5.2. Exosomes for Curcumin Delivery

To sum things up, to increase curcumin solubility as well as bioavailability and thereby enhancing curcumin therapeutic efficacy, an optimized drug delivery system may be needed. For this purpose, curcumin can be encapsulated into liposomes, cyclodextrin, polymeric NPs, microspheres, hydrogels, and exosomes [38,170,173]. To achieve proper function and performance of the liposome-based delivery system, various components must be appropriately selected and controlled [37]. It is noteworthy that an exosome can carry multiple drugs to adjust the activity of various pathways [38]. 

The protective environment of curcumin provided by exosomes makes exosome appropriate carrier for oral administration [174]. The use of exosomal curcumin may have benefits for curcumin function, as well [38]. Encapsulated curcumin into exosomes is stable compared to the free curcumin and can protect curcumin from the environment of the human digestive system and protect the intestinal epithelium [174]. Curcumin can be encapsulated in two layers of lipid exosomes, which results in the protection of curcumin from damage. Theoretically, circulating exosomes due to endogenous origins and a particular superficial composition should be more stable than other synthetic polymer-based NPs, such as liposomes [175]. It appears that the encapsulation of curcumin into exosomes significantly increases the solubility, bioavailability, and stability of curcumin [38]. 

In recent years, exosome delivery as a promising approach for carrying therapeutics across the BBB to the central nervous system (CNS) has been an increasing interest among researchers. Kalani et al. demonstrated that curcumin-primed and curcumin-loaded exosomes (40–200 nm) had neuroprotective effects due to their anti-lipidemic, anti-oxidative, and anti-inflammatory nature [176]. Furthermore, curcumin-primed exosomes (117 ± 10 nm) prepared for drug delivery into CNS in vitro and in vivo. Effective BBB transport achieved via receptor-mediated transcytosis, in which active targeting of ICAM-1 proteins was provided by the LFA-1 functionalization of exosomes. Results indicated that curcumin-primed exosomes prevent neural death and improve Alzheimer’s disease symptoms owing to an inhibition of Tau protein phosphorylation through the AKT/GSK-3β pathway [177]. Intranasal administration of curcumin encapsulated into exosomes causes NMDAR1 expression accompanied by the reduction of edema, infarct size, astrogliosis, and vascular inflammation as well as tight and adherent-junctions recovery and NeuN positive neurons restoration after an ischemia-reperfusion injury in the mouse model. Potent therapeutic effects of curcumin encapsulated into exosomes are in part related to the numerous secreted paracrine factors derived from mesenchymal stem cells along with extraordinary curcumin anti-inflammation and neuroprotective potentials [170].

Bovine milk is a rich source of exosomes for carrying therapeutics like curcumin. Curcumin-loaded into milk exosomes (30–100 nm) possess higher bioavailability, aqueous solubility, and stability as well as easier crossing through the intestinal barrier in compare with free curcumin [174]. Interestingly, oral delivery of curcumin via milk-derived exosomes (~93 nm) showed a strong inhibitory effect in mice bearing cervical tumor xenograft [178]. However, curcumin-exosome NPs exhibit higher solubility and antioxidant efficacy (~41 nm) in comparison with free curcumin by using fluorescence tracking analysis [179].

## 6. Co-Polymers

In order to improve bioavailability and hydrophilicity of curcumin, colloidal drug delivery systems are used to solubilize medicines. There has been interest in the use of polymeric nano-particulate delivery systems composed of biocompatible, biodegradable, amphiphilic diblock copolymers for the intravenous administration of hydrophobic compounds [180]. Polymer-based nano-carriers demonstrate good potential as an anti-cancer therapeutics [181]. The effect may be due to their favorable properties like small size, enormous biocompatibility and biodegradability, high stability, prolonged circulation time in the bloodstream, improved drug loading capacity, as well as tendency for easy chemical modifications [181,182].

Biochemical reactions occur in the micrometer or sub-micrometer-sized environments. The various categories of molecules such as hydrophilic drugs or enzymes can be applied to design a highly efficient alternations [183]. Depending on the diblock composition and addition to aqueous media, these copolymers self-gathered to form structures in a nano-sized termed micelles [180]. Polymeric micelles defined as core-shell nanoparticles that may be used as appropriate carriers of biologically active compounds for different medical applications [182]. Poly (ethylene glycol) (PEG) and poly (e-caprolactone) (PCL) can be useful in the construction of polymeric carriers. PEG that has been approved by the United States Food and Drug Administration (FDA) is a hydrophilic, non-toxic polymer, and is used for medical purposes. It exhibits low protein adsorption and cell adhesion properties [184]. The novel chitosan-coated XGO-b-PCL nanoparticles are another example of a better strategy to improve the therapeutic efficacy of hydrophobic drugs [185].

Block copolymer nano-carriers have been used for the delivery of curcumin [182] for example cationic PDMAEMA–PCL–PDMAEMA micelles were developed and provide sustained release and modified antioxidant activity of curcumin. Yoncheva and et al. studied the effect of these particles on K562 and U266 cells and found that cytotoxic capacity of micellar curcumin is better than the effect of free curcumin at lower concentrations [181]. In another study, curcumin was incorporated into the core of PEO113-b-PnBA235-b-PAA14 micelles. The release profiles of curcumin from micelles showed sustained release without burst effect [182]. Additionally, Tabatabaei et al. prepared curcumin loaded PLGA-PEG NPs and showed that the anti-cancer efficacy of curcumin triblock copolymer NPs (70–300 nm, encapsulation efficacy 84.5%) is significantly higher than curcumin in MCF-7 human breast cells [186]. PLGA a polymer consisting of PGA and PLA is considered as a copolymer and used for drug delivery due to its non-significant toxicity and modulation of hydrophilicity/hydrophobicity. Hu et al. prepared a curcumin-PLGA NP and investigated the inhibition of opioid tolerance in mice treated with morphine. Interestingly, administration of curcumin-PLGA NP resulted in 11–33 fold decrease in morphine dose in mice in part through the inhibition of activity of Ca2+/calmodulin-dependent protein kinase II α [19]. Furthermore, Shen et al. prepared three types of NP including curcumin-PLGA, Curcumin- PEG-b-PLA and the combination of curcumin-PLGA-PEG-b-PLA to investigate attenuation of morphine tolerance in mice. The particle size of all NPs was similar and around 150 nm. Results of tail-flick in mice were stronger than with non-formulated curcumin [187,188]. These findings are very important especially for the diseases associated with chronic pain. 

Moreover, encapsulation of curcumin into a polymer based and other abovementioned materials can enhance the bioavailability of curcumin and its drug efficacy. It appears that there are some conflicting data on the correlation between the particle size and drug bioavailability. For example, Sun et al. reported that NPs at the range of 700–120 nm enhanced bioavailability compared to the suspension form while there was no significant between the NPs at the range of 700 to 120 nm. Otherwise, bioavailability significantly enhance when the particle size drops to 80 nm [189]. While, Vrana et al. demonstrated that there is not a significant correlation between the particle size and cyclosporine A formulations [190]. If it is considered that water solubility (hydrophobicity) is one the most critical factors in drug bioavailability, therefore it might be said that the contact angle of a nanocarrier is a critical marker to predict drug bioavailability and eventually this hypothesis may explain the controversy data on the correlation of particle size and bioavailability. The important point is that particle size and surface charge are just two markers in the final fate of contact angle and there are some other parameters such as roughness, morphology etc., that may affect contact angle. 

## 7. Targeted Delivery

Targeted delivery can be divided into active and passive targeted delivery in which active targeted delivery is usually through some antigen or receptor on/in targeted cells while passive delivery is mediated via enhanced permeability and retention effect (EPR) mechanism. There are some molecules that can act as a ligand for targeting drug delivery such as antibodies fragment and monoclonal antibodies, aptamers, folic acid and etc. (Table 2) [37,191,192]. Curcumin as an anti-inflammatory agent has been widely used a potential drug in cancer therapy. Breast cancer cells overexpresses sialic acid in cell membrane and Kundu et al. conjugated curcumin to phenyl boronic acid (PBA) and ZnO NPs. Notably, Curcumin-PBA-ZnO NP (40 nm) can significantly decrease tumor growth in mice bearing Ehrlich ascites carcinoma (EAC) tumor [193]. In another study, gold nanorods and curcumin were loaded into a PLGA-b-PEG co-polymer NPs (~137 nm). The anti-cancer efficacy of the nanocarrier with and without curcumin was investigated. The results disclosed the retention of both gold-PLGA-b-PEG NPs (with and without circumin) in cancer cells located in esophageal mucosa, but not in normal esophageal mucosa in a Barrett’s associated animal model upon NIR irradiation [194]. Another example of targeted delivery is related to folic acid attached to curcumin-albumin-Bi2S3 NPs. The combination of chemotherapy and radiotherapy led to an enhanced efficacy in an animal model of tumor [195].

There are some reports related to conjugation of folate to NPs as a cargo for anti-cancer delivery. Another example of targeted curcumin delivery is related to the conjugation of curcumin to hyaluronic acid (HA) and folic acid. Both HA (CD 44 receptor) and folic acid are over-expressed in cancer cells. However, their attachments to gold NPs, HA, and PEG enhance cancer cell toxicity and drug circumstance and up-take, respectively. Interestingly, the final particle size was approximately 120 nm [199]. In fact, folate conjugation enhances active targeting through increased transportation of cargo using endocytosis into the cell. For example, Thulasidasan et al. conjugated folate on the surface of PLGA-PEG loaded curcumin NP and evaluated the NP in combination with paclitaxel and compared it with liposome. The results showed synergistic cytotoxicity in Hela cancer cells along with enhanced retention time in cervix tissue of Swiss albino mice [200]. Huong et al. attached folic acid to a NP containing magnetic NP, curcumin and coated it with O-carboxylmethylchitosan. Overall, the outcomes indicated that targeted delivery system can enhance bio-distribution in mice bearing a sarcoma-180 solid tumor. Notwithstanding, magnetic NPs under magnetic field can induce heat and trigger cell death mechanisms especially in cancer cells owing to two reasons: (1) Cancer cells are more susceptible to the high temperature (42 °C) compared to the normal cells, and (2) Passive targeting of magnetic NPs owing to their particle size [201]. Song et al. conjugated folate on the surface of albumin NPs loaded curcumin as a carrier (~165 nm, −27.3 mV) for cancer therapy in mice induced by HT29 cells subcutaneously. The results showed a higher anti-tumor efficacy of water soluble curcumin NPs in part due to the inhibition of drug metabolism that resulted in an enhanced anti-tumor efficacy [188]. 

As mentioned earlier, aptamers also function as a ligand for targeting delivery on the surface of NPs. Lei et al. conjugated RNA aptamers for epithelial cell adhesion molecule (EpCAM) protein on the surface of PLGA-lecithin-PEG NPs (less than 100 nm) containing of curcumin. They investigated its efficacy on colorectal adenocarcinoma cells. The half-life and retention time of curcumin NPs increased as compared to the free curcumin, 6 and 3-fold, respectively. However, the cancer cell cytotoxicity and bioavailability of cargo was significantly enhanced as compared to the curcumin [202]. Moreover, Huang et al. used galactosylation as a potential ligand for cell targeting. They galactosylated albumin NPs encapsulated curcumin as carrier (116.24 nm) for HepG2 cancer cell therapy. It was noted that galactose selectively bound to the receptors on the surface of cancer cells and inhibited NF-κB activation and cell migration, thus resulting in enhanced anti-tumor efficacy [196].

## 8. Conclusions and Future Trends

Curcumin holds a great promise among the nutraceuticals due to its pleiotropic biological activities. However, its poor solubility and bioavailability may limit its application in the clinic. To overcome these drawbacks, it seems that encapsulation into specific nanocarriers can be of great interest and enhance its applications. Here in this review, we have discussed different types of biopolymers and biological carriers in different forms that can be used for curcumin delivery. For this purpose, at first, we described protein-based biopolymers which are biocompatible carriers with the ability to form different types of nanocarriers. Then different kinds of polysaccharide biopolymers are discussed along with their characteristic features like biocompatibility, bioavailability, low cost, and biodegradability. At the end, the biological carriers with specific focus on exosomes, their excellent properties, and preparation methods have been highlighted. Overall, the encapsulation of curcumin into these various carriers may lead to a significant enhancement of its various anti-cancer activities. Although the biocompatibility and anti-cancer efficacy of the above-mentioned nanocarriers have been partially validated in different models, however, further in vivo and clinical studies are needed to facilitate their safe administration in cancer patients.

## Figures and Tables

**Table 1:** A list of different curcumin nanocarriers with their characteristics and applications under in vitro and in vivo settings.

Polymer	Size	Zeta Potential	LC or EE	Cell Line/Animal Model	Advantages	Refs.
BSA@CUR NPs	92.59 ± 16.75 nm	−9.19 mV	18.3%	MCF-7 cells	Increased therapeutic efficacy	[77]
Curcumin in BSA-dextran NP	115 nm		2.8%	Caco-2 cells	Better stability Improve the cellular antioxidant activity of curcumin	[91]
Curcumin cross-linked HSA NPs	125 nm	−12.36 ± 0.73 to −10.88 ± 0.6 mV	was dependent on the particle size	A549 cells	Improved cellular uptake Increased the cytotoxicity	[92]
Curcumin-loaded zein NPs	66 nm	+17.1 mV	7.3 ± 0.1%	GIT model	May be useful for application in functional foods or beverages	[93]
Curcumin-zein/rhamnolipid complex	77.29 nm	−31 mV To +3 mV	EE: 98.05%	In vitro simulated gastrointestinal tract	Protect hydrophobic bioactive compounds	[94]
Pectin-coated CZ NPs	250 nm to 600 nm	−45 to −50 mV	5%	Simulated gastrointestinal digestive condition	Enhanced antioxidant activity in an aqueous environment	[95]
Curcumin-loaded zein NPs with (SC) and (SA)	190 nm	17 mV to 19.8 mV	EE: 36.10% to 76.06%		Improving the water solubility Improving photochemical stability improving antioxidant activity	[96]
Curcumin-loaded silk fibroin NPs	155 nm to 170 nm	−45 mV	EE: 50%	Kelly Cells	Higher efficacy in cytotoxicity	[7]
Curcumin plus SFNs	71 ± 10 nm		1.50 ± 0.11 to 11.40 ± 0.76	In vitro model of osteoarthritis	Exhibited a synergistic antioxidant effect Improve cyto- and hemo-compatibility	[97]
CUR-loaded silk NPs	229 nm to 2286 nm	−17.8 nm to −18.9 mV	22 to 41%	Rats	Longer plasma circulation time	[98]
CUR Loaded RBA−CS NPs	778 nm	Negative	EE: 93.56%	Caco-2 cells	A great potential application for hydrophobic active agent delivery	[99]
Zein-HA NPs	186.4 nm	–35.2 to −28.7 mV	3.66%	Simulate gastrointestinal digestion	Better stability of anti-light degradation, and control release	[100]
SSPS NPs	200 nm to 300 nm		EE: 90%	HCT116 and MCF-7 cells	Improved activity Improvement in the anti-proliferative activity	[101]
Cur-ACRU/CS NPs	200 nm to 450 nm	+15 mV	5.4%	Caco-2 cells	Improved permeability efficiency of free curcumin	[102]
Cur-Chitosan NPs	167 nm to 251 nm	+ 18.1 to + 20.2 mV	EE: 80%	HaCaT cells	Superior drug release Enhanced transdermal permeation of curcumin A superior percentage of cell viability	[103]
CDG-CANPs	215 nm	−24.1 mV	27%	Caco-2 cells	Improvement of physicochemical stabilities, digestibility, bioaccessibility and cellular uptake	[104]
CUR-AlgNP	100-600 nm	−36.0± 0.4	EE: 68.3%	HeLa and H9c2	Kills the cancer cell lines at lower concentrations	[105]
Cur-CS/Alg NPs	199 nm to 1120 nm	−30.8 mV to −10.8 mV	0% to 27.4%	HaCaT cells	Improved the cellular uptake of curcumin	[106]
Starch NPs	< 250 nm	−30 mV	EE: 80%	Simulated gastric and intestinal fluids	Higher encapsulation efficiency	[107]
OSA starch loaded nano curcumin	10 nm to 50 nm			HeLa cells	Anti-cancer potential Significant enhancement in cellular uptake Increase bioavailability More controlled release	[108]
Curcumin-load film	159 ± 31 nm in length and 2 nm in width.			Rat	Improved the regeneration of hair follicles And sebaceous glands of the skin Attenuated the bacterial growth	[109]
Cur- NLCs	500 nm		EE~58.8 ± 3.5	Mouse	Reducing the pro-inflammatory cytokine levels in the skin	[110]
ANC NPs	≤150 nm	-31.2 ± 3.66 mV	EE > 90%	L929 and MCF-7 cells	Inhibit microbial growth Prevent preferential killing of cancer cells compared to normal cells	[111]
WPI-Lac/EGCG NPs	110 nm	27 mV			Better protective effect on the breakdown of curcumin in Pickering emulsions More even droplet distribution Greater thermal stability Higher curcumin percentage retention	[112]
CUR-Loaded Gel-mPEG Nanogels	147 ± 5.2 nm	−12.8 ± 0.6	7.9 ± 0.2%,	HeLa cells	Improved solubility Enhanced therapeutic efficacy	[113]
Curcumin-loaded BSA NPs	150 nm	Negative	EE: 45%	Murine melanoma model	Increase in survival rate associated with a reduction in tumor size	[114]
Curcumin loading EWP	59.25 nm to 431.3 nm	>+30 mV	11.2 mg/g		Protect the antioxidant activity of encapsulated curcumin	[115]
Curcumin-PECs	264.0 ± 3.1 nm		EE: 53%	HCT116 cells	Induced cell cycle arrest Exhibited cytotoxic effect	[116]

**Table 2:** Targeted curcumin delivery using biopolymers-based nanoparticles.

Polymer	The Route of Targeting	Size	Zeta Potential	LC or EE	Cell Line/Animal Model	Advantages	Refs.
F-CUR-HSANPs	Folate	165.6 ± 15.7 nm	−27.3 ± 4.2 mV	EE:88.7% ± 4.8%	Murine colon cancer model	Maintained sustained release, and a faster release of CM compare to the unconjugated NPs	[188]
Apt-HSA/CCM NP	Aptamer to target HER-2 positive cells	281.1 nm	−33.3 ± 2.5mV	3.4%	SK-BR3 cells	Higher toxicity	[120]
Gal-BSA-Cur NPs	Galactosylation to target asialoglycoprotein receptor (ASGPR) overexpressed on hepatocellular carcinoma (HCC) cells	116.24 nm	−14.12 ± 1.81	EE:55.47% ± 0.45%	HCC cell line	Enhanced the internalization ability of drug compared with BSA NPs-loaded curcumin	[196]
Zein and HA for the co-delivery of curcumin and quercetagetin	HA	231 nm	−30.5 mV	2.5%	simulated gastrointestinal tract conditions	Improve oral bioavailability	[122]
Curcumin loaded magnetic silk fibroin core–shell NPs	Magnetic NP	30 nm to 250 nm		LC: 8.4%	MDA-MB-231 cells	Enhanced growth inhibition	[128]
Bi2S3@BSA-FA-CUR	Folic acid	170.9 nm	−23.2 mV	LC:10 ± 1.51%	The mouse breast carcinoma cell line, Murine breast cancer model	Enhanced the efficacy of chemoradiation therapy	[195]
magnetic alginate/chitosan layer-by-layer nanoparticles (MACPs)	Fe3O4 NPs	172 nm to 199 nm		EE: 49.2%	MDA-MB-231 breast cancer cells, HDF cells	The sustained release profiles, enhanced uptake efficiency and cytotoxicity to cancer cells	[197]
folic acid tagged aminated starch/ZnO coated iron oxide nanoparticles as targeted curcumin delivery system	Fe3O4 NPs	31.2 ± 2	42.9 ± 0.03	EE: 76.8 ± 0.04%	HepG2 and MCF7 cell lines	Enhanced the uptake by HepG2 cells	[198]
Cur loaded MnFe2O4–CMC	Fe2O4 NPs	35 nm			MCF7 and HeLa cells	Enhanced the therapeutic efficacy	[156]

## Abbreviations

VEGF	Vascular endothelial growth factor
HAS	human serum albumin
BSA	bovine serum albumin
NPs	nanoparticles
HER2	human epidermal growth factor receptor 2
HA	hyaluronic acid
Apt-HSA/CCM	aptamer-decorated curcumin-loaded human serum albumin
SSPS	soluble soybean polysaccharide
Cur-ACRU/CS	curcumin-loaded acylated cruciferin/charged chitosan
CDG-CANPs	curcumin diethyl diglutarate-loaded Chitosan/alginate NPs
CUR-AlgNP	curcumin loaded alginate NP
Cur-CS/Alg NPs	curcumin-loaded chitosan/alginate NPs
CMC	carboxymethyl cellulose
Cur-NLCs	curcumin loaded nanostructured lipid carriers
ANC	aminated nanocellulose
EWP	egg white protein
PECs	polyelectrolyte complexes
SC	sodium caseinate
SA	sodium alginate
PEG	poly (ethylene glycol)
PCL	poly (e-caprolactone)
FDA	U.S. Food and Drug administration
